# Response to letters to the editor regarding ‘feasibility of radiation therapist–performed treatment reviews’

**DOI:** 10.1002/jmrs.27

**Published:** 2013-11-20

**Authors:** Clare M Monk, Tony Neil Smith

**Affiliations:** Department of Radiation Oncology, Calvary Mater, Newcastle NSW, Australia; Department of Rural Health, University of Newcastle NSW, Australia

Re: Monk CM, Wrightson SJ, Smith TN. An exploration of the feasibility of radiation therapist participation in treatment reviews. *J Med Rad Sci* 2013; **60**(3): 100–7

Thank you for the opportunity to respond to the letters to the editor following the publication of our article about the feasibility of implementing radiation therapist treatment reviews at the Calvary Mater Newcastle.^1^ We are pleased that the article stimulated discussion and a response from two very senior and expert radiation therapists.

In response to the letter from Jill Harris, we agree with what is said. In relation to the question of ‘whether substantial interprofessional communication occurred prior to the study’, the answer is definitely ‘no’. If we had had substantial discussions with any of the staff members who were asked to respond to the survey it would have biased the study and, of course, we did not want to do that. In reality, there will have been some discussion between the staff members, from casual tea room conversations to conferences and other professional forums. In some ways these conversations could have confounded the study; however, not knowing the content and tone of these discussions it is difficult to say what affect, if any, they had on the results.

The most important point, as Jill rightly reaffirms, is that opinions are going to vary and that this will always be a local confounder in attempts to introduce models of advanced practice. Clearly, the best option would be to have national agreement on advanced practice between the principal professional bodies involved, but this seems to be some way off in spite of the inroads made by the Inter-Professional Advisory Team^2^ in the recent past.

Jenny Cox also makes some valid points and reaffirms some of the findings of our study. In particular, she is supportive of the finding that breast and prostate cancer treatments have low levels of medical intervention and that these treatment review clinics should be primary targets for radiation therapist advanced practice. We also agree with Jenny that radiation therapists have the necessary attributes to take on this role; however, we make the equally strong point that there is a lot of work still to be done in terms of gaining the support of radiation oncologists and providing radiation therapists with the necessary educational platform from which to take on advanced practice roles. The same is true in diagnostic imaging.

We think that one of the benefits of this study is that it provides evidence that opinions and attitudes about radiation therapist (or diagnostic radiographer) advanced practice are likely to vary substantially from department to department. While it is clear that models of radiation therapist treatment review can be successfully implemented in one department, as both Jill and Jenny point out, this does not mean that implementation is possible universally. We argue that there are significant local issues that have to be dealt with on a case-by-case basis and that there is an important need for the professional bodies representing both oncologists and radiation therapists to come together and negotiate role boundaries with a view to having a national policy framework that might inform the development of local practice models. We further advocate for qualitative research to take place in the near future to define the practice boundaries in all areas of advanced practice.
